# Association of low blood pressure with suicidal ideation: a cross-sectional study of 10,708 adults with normal or low blood pressure in Korea

**DOI:** 10.1186/s12889-018-5106-5

**Published:** 2018-03-01

**Authors:** Kyung-in Joung, Sung-il Cho

**Affiliations:** 1 Department of Public Health Science, Graduate School of Public Health, Seoul National University, Seoul, South Korea; 2 Department of Public Health Science, Graduate School of Public Health and Institute of Health and Environment, Seoul National University, Seoul, South Korea

**Keywords:** Low blood pressure, Hypotension, Suicidal ideation, Depression, Psychological symptoms, Korean National Health and Nutrition Examination Survey

## Abstract

**Background:**

Whether constitutional low blood pressure (BP) causes substantive health problems has been controversial, and subjects with hypotension exhibit a range of symptoms, from mild typical conditions such as tiredness and dizziness to more specific psychological conditions and even cognitive disorders. This study investigated whether low BP is associated with suicidal ideation in the general population.

**Methods:**

Four years of data from the 2010–2013 Korean National Health and Nutrition Examination Survey were used. Among the 23,163 participants, aged 19–101 years, 10,708 with normal or low BP were included in the analysis of the association between low BP and suicidal ideation. The criterion used for low BP was systolic BP (SBP) < 100 mmHg, and in comparative analyses, the criteria used for low BP were SBP < 110, < 95, and < 90 mmHg. The association of prehypertension or hypertension with suicidal ideation was also examined. Suicidal ideation was assessed by a questionnaire.

**Results:**

Compared with the normotensive reference group, the odds ratios (ORs) for suicidal ideation were significantly higher in the three hypotensive groups after adjusting for sex, age, body mass index, total cholesterol level, household income, educational level, marital status, current smoking status, alcohol intake, and the interaction between sex and age (OR = 1.29, 95% confidence interval [CI], 1.08 to 1.55; OR = 1.44, 95% CI, 1.14 to 1.82; and OR = 1.71, 95% CI, 1.11 to 2.62 for SBP < 100, SBP < 95, and SBP < 90 mmHg, respectively). Adding the clinical morbidities of diabetes mellitus, stroke, myocardial infarction/angina pectoris, and depression as covariates had little effect on the strength of the associations (OR = 1.25, 95% CI, 1.04 to 1.50; OR = 1.43, 95% CI, 1.13 to 1.81; and OR = 1.74, 95% CI, 1.14 to 2.68 for SBP < 100, < 95, and < 90 mmHg, respectively).

**Conclusions:**

Low SBP showed an association with suicidal ideation in the general Korean population. The association was significant for low BP, defined as a SBP < 100 mmHg, and the strength of the association increased as the criteria for low BP increased in strictness.

**Electronic supplementary material:**

The online version of this article (10.1186/s12889-018-5106-5) contains supplementary material, which is available to authorized users.

## Background

Most studies on blood pressure (BP) have focused on high BP, whereas studies on low BP are rare [1]. Unlike hypertension, constitutional low BP is not considered to be related to serious illness or death [1], and it is widely accepted that ‘the lower the BP the better’ [2]. However, most existing studies on whether constitutional low BP (hereinafter referred as low BP) can lead to adverse health outcomes have shown that low BP is associated with physical or mental symptoms or disease [3, 4], except for a few studies [5, 6]. In a large cross-sectional study, Wessely [3] showed a relationship between systolic BP (SBP) and self-reported tiredness and feeling faint. Barret-Connor et al. [4] reported that men with a diastolic BP (DBP) < 75 mmHg had a significantly higher rate of depression. More recently, two large-scale prevalence studies in older individuals also suggested that low BP is associated with depression [7, 8]. Hildrum et al. [9] reported that low BP is associated with depression, anxiety, and comorbidities of anxiety and depression in a cross-sectional study of 60,799 patients in the general population. Recent studies on low BP aimed mainly to identify correlations with neurological aspects. Although the results are mixed, a number of studies have suggested that low BP is associated with cognitive impairment, dementia, or Alzheimer’s disease [10–12]. Qiu et al. [11] showed that extremely low DBP (≤65 vs. 66–90 mmHg) had an adjusted relative risk of 1.7 (95% confidence interval [CI], 1.1 to 2.4) for Alzheimer’s disease and 1.5 (95% CI, 1.0 to 2.1) for dementia. A 13-year prospective cohort study reported that both high and low BP are associated with poorer cognitive performance in elderly African-Americans [12].

It is biologically plausible that hypotension is associated with mental and neurological health problems. Many recent studies that have shown a relationship between low BP and cognitive impairment suggested low cerebral perfusion as the biological mechanism. Studies that have identified a link between low BP and depression also suggested that cerebral vascular perfusion is a contributing factor, and that overexpression of neuropeptide Y observed in both depression and low BP may mediate this relationship [13, 14].

Suicide is a leading cause of death worldwide, and over 800,000 people die from suicide every year [15]. As suicidal ideation is a key stage in the pathway leading to suicide, a greater understanding of its risk factors could help in the development of suicide prevention strategies [16, 17].

It is well established that mental disorders, such as depression, amplify suicidal ideation and suicide [18, 19], whereas the role of physical conditions is controversial [20]. In a systematic review conducted in 2016, dysfunction, malignant tumor diseases, neurological disorders, male genital disorders, arthritis/arthrosis, chronic obstructive pulmonary disease, and liver disease were consistently associated with suicidal behavior, but cardiovascular disease showed little association. However, in a large cross-national study, myocardial infarction (MI) and stroke, but not cancer, were associated with suicidal ideation [21]. Most population and register-based studies reported no association between diabetes mellitus (DM) and suicidal behavior [20].

Several studies have examined the relationship between BP and suicidal ideation, but all were concerned with high BP. Scott et al. [21] suggested an increased risk of suicidal ideation in a high BP group, after adjusting for other types of physical conditions and mental disorders, although the risk was marginally significant (odds ratio [OR] = 1.3, 95% CI, 0.89 to 1.19). In contrast, some studies have shown that hypertension is not associated with suicidal ideation in older individuals [22, 23].

Despite the suspicion that low BP is associated with depression, which is a potent risk factor for suicidal ideation, no studies have investigated the association between low BP and suicidal ideation. Hirsch et al. [18] suggested that trait positive affect is an important independent contributor for reducing suicidal ideation. Subsequently, Kim et al. [7] showed that low BP is related to depression, specifically to a low positive affect. These two studies make it even more plausible that the risk of suicidal ideation in individuals with low BP is high.

Although the majority of studies on hypotension and mental health have been limited to older populations, the prevalence of hypotension is much higher in young adults [3]. In addition, the clinical interventions for low BP differ between English-speaking countries and continental European countries. Therefore, studies on the health impacts of hypotension are needed for populations of young adults from various countries, ethnicities, and races.

The purpose of this study was to investigate the association between low BP and suicidal ideation among adults aged > 19 years in Korea. Low BP was analyzed according to different SBP criteria, to determine whether there is a quantitative relationship in which the risk of suicidal ideation increases with decreasing cut-off levels for low BP.

## Methods

### Study sample

Four years of data from Korean National Health and Nutrition Examination Survey (KNHANES; 2010–2013) were used. The KNHANES is a well-validated, nationwide, large-scale survey that is conducted to understand the health and nutritional state of Koreans. It has produced statistical resources necessary for establishing and evaluating health policies, based on Article 16 of Law for the Promotion of the Nation’s Health. KNHANES data are publicly available and de-identified. The target population of KNHANES comprises noninstitutionalized Korean citizens residing in Korea.

Data collected from the health survey include a household and health interview, a health behavior survey, and a medical examination. KNHANES used the population and housing census data employing the sample extraction frame and resident registration population to extract a representative sample of Korean citizens. The sampling plan follows a multi-stage clustered probability design. More details on KNHANES are described on the KNHANES homepage (https://knhanes.cdc.go.kr/knhanes/main.do). Participants aged > 19 years with measured BP data who responded to the question on suicidal ideation were included. Those with an unknown antihypertensive status were excluded.

### Measures

The BP measurements were performed by four nurses within the expert examination execution team. Since 2010, quality management for BP measurements has been further strengthened, and a BP measurement examiner certification system was introduced. After taking three measurements, the first was excluded, and the average of the second and third was used as the final BP. The criterion for low BP was SBP < 100 mmHg, and the criteria for low BP were SBP < 110, < 95, and < 90 mmHg for comparative analyses between groups based on a high or low BP cut-off. BPs other than low BPs were classified as normotensive (SBP < 120 mmHg and DBP < 80 mmHg), prehypertensive (120 ≤ SBP < 140 mmHg or 80 ≤ DBP < 90 mmHg), or hypertensive (SBP ≥ 140 mmHg or DBP ≥ 90 mmHg), following the American Heart Association classification. Those who took antihypertensive drugs were classified in the hypertensive group regardless of measured BP values.

Suicidal ideation was identified in respondents by asking the following questions: “Have you ever felt inclined to commit suicide over the last year?” (2010–2012), and “Have you ever considered suicide seriously over the last year?” (2013). Because we did not evaluate suicidal ideation by year, integrating data from the slightly different questions seemed reasonable.

To identify and control for confounding variables, age, body mass index (BMI), and total cholesterol level were included as continuous variables, while sex, household income, educational level, marital status, current smoking status, alcohol intake, DM, stroke, MI/angina pectoris, and depression were included as categorical variables. Household income and education were categorized into four levels. Those who had smoked at least 100 cigarettes over their entire life and were currently smoking were classified as current smokers. Alcohol intake was classified into three levels based on drinking frequency. Clinical morbidities for DM, stroke, MI/angina pectoris, and depression were classified into those who currently had a morbidity and those who did not or had never been diagnosed.

### Statistical analysis

Basic demographic characteristics and medical conditions were compared between the suicidal ideation group and reference group using χ2 tests. Multivariate logistic regression was used to assess the association between low BP and suicidal ideation. Multiple logistic regression was also used to identify the risk of suicidal ideation in the prehypertensive or hypertensive groups (supplementary analyses). Multiple logistic regression was calculated using sample weights assigned to the sample participants. The sample weights were prepared for sample participants to represent the Korean population by accounting for the complex survey design, non-responses to the survey, and post-stratification [24].

All analyses were performed using ‘R’ software (‘R’ version. 3.2.3, R Foundation for Statistical Computing, Vienna, Austria).

Ethics approval to conduct the study was received from the SNU Bioethics Deliberation Committee, Seoul National University.

## Results

### Participants

The study included 24,238 participants aged > 19 years. Those participants with missing BP values or with an unknown antihypertensive usage status were removed, leaving 23,163 subjects. Of these, the number of participants belonging to the normotensive and hypotensive groups was 10,733. For the analysis of association between low BP and suicidal ideation, 10,708 subjects were included after excluding non-responders to the questions on suicidal ideation. Those with a SBP < 70 mmHg and DBP < 30 mmHg were deemed outliers, but none of the subjects had such levels.

### Descriptive data

When the criterion for hypotension was SBP < 100 mmHg, 2569 subjects were (24.0%) classified into this group, and the basic characteristics of this group compared with those of the normotensive group are shown in Table 1. When a criterion of SBP < 110 mmHg was used, 7238 subjects (67.6%) were classified into the hypotensive group. Low BP was more common in women, younger participants, underweight participants, and those with lower total cholesterol levels. Differences were also found in household income, educational level, and marital status. Fewer smokers and alcohol drinkers were seen in the low BP group. Clinical morbidities were also different between the two groups, but the sample size of the patients was very small, except that of those with DM (Table 1).

**Table 1:** Basic characteristics of the hypotensive group compared with the normotensive group (2010–2013)

Characteristic	Reference group ^a^	Low blood pressure ^b^
	*n* = 8139 (76.0%)	*n* = 2569 (24.0%)
Sex		
Men	3143 (86.0%)	511 (14.0%)
Women	4996 (70.8%)	2058 (29.2%)
Age (mean), years	43.5 ± 15.0	38.7 ± 12.9
Age group		
19~ 29	1531 (70.0%)	655 (30.0%)
30~ 39	2165 (71.3%)	870 (28.7%)
40~ 49	1754 (75.8%)	560 (24.2%)
50~ 59	1375 (82.7%)	287 (17.3%)
60~ 69	762 (85.9%)	125 (14.4%)
≥ 70	552 (88.5%)	72 (11.5%)
SBP, mmHg	108.8 ± 5.4	94.6 ± 4.0
DBP, mmHg	70.6 ± 5.6	63.2 ± 5.9
Mean Body Mass Index	22.9 ± 3.2	21.7 ± 2.8
Body Mass Index (kg/m^2^)		
< 18.5	508 (65.0%)	274 (35.0%)
≥ 18.5, < 25	5718 (74.3%)	1978 (25.7%)
≥ 25	1899 (86.0%)	310 (14.0%)
Hemoglobin, g/dl	13.8 ± 1.6	13.2 ± 1.4
Total cholesterol level, mg/ml	186.3 ± 34.9	177.8 ± 32.6
Household income		
Low	1958 (77.6%)	566 (22.4%)
Low to intermediate	2005 (77.0%)	599 (23.0%)
Intermediate to high	2020 (75.7%)	650 (24.3%)
High	2075 (73.8%)	738 (26.2%)
Educational level		
Elementary school	1133 (86.4%)	178 (13.6%)
Middle school	695 (83.7%)	135 (16.3%)
High school	3215 (76.1%)	1010 (23.9%)
College/University	3094 (71.3%)	1246 (28.7%)
Marital status		
Single	1892 (77.3%)	556 (22.7%)
Married	5771 (76.2%)	1803 (23.8%)
Rejection response	469 (69.5%)	206 (30.5%)
Current smoking status		
Yes	1743 (82.7%)	364 (17.3%)
No	6395 (74.4%)	2204 (25.6%)
Alcohol intake		
1 drink</month	2812 (72.4%)	1072 (27.6%)
1~ 4 drink /month	3026 (76.0%)	957 (24.0%)
≥ 2~ 3 drink /week	1473 (82.8%)	307 (17.2%)
Diabetes mellitus		
Yes	273 (84.5%)	50 (15.5%)
No	7866 (75.7%)	2519 (24.3%)
Stroke		
Yes	7 (43.8%)	9 (56.3%)
No	8102 (76.0%)	2560 (24.0%)
MI/Angina pectoris		
Yes	95 (84.1%)	18 (15.9%)
No	8044 (75.9%)	2551 (24.1%)
Depression		
Yes	125 (74.4%)	43 (25.6%)
No	8014 (76.0%)	2526 (24.0%)

*SBP* Systolic Blood Pressure, *DBP* Diastolic Blood Pressure, *MI* Myocardial infarction

^a^Reference group, Normal blood pressure, 100 ≤ SBP < 120 mmHg & DBP < 80 mmHg

^b^Low blood pressure, SBP < 100 mmHg

The baseline characteristics of the participants with suicidal ideation are shown in Table 2. The proportion of subjects with suicidal ideation was 11.2% (1199 subjects). Suicidal ideation was more common in women than men (12.9% vs. 7.8%) and was most prevalent in the oldest age group (age ≥ 70 years, 20.8%). When the lower SBP cut-off was used to define low BP, the proportion of subjects with suicidal ideation increased (11.2%, 12.5%, 13.7%, 16.6% for SBP < 110, SBP < 100, SBP < 95, and SBP < 90 mmHg respectively). In addition, suicidal ideation was more common among adults with a lower household income and a lower education level. The higher prevalence of suicidal ideation was observed among those with depressed mood, stress perception, and certain diseases. (Table 2).

**Table 2:** Characteristics of the study population by suicidal ideation (2010–2013)

	Reference ^a^	Suicidal ideation	*p-*value
	9509 (88.8%)	1199 (11.2%)	
Sex			0.00
Men	3368 (92.2%)	286 (7.8%)	
Women	6141 (87.1%)	913 (12.9%)	
Age (mean), years	42.2 ± 14.4	44.1 ± 16.7	0.00
Age group			0.00
19~ 29	1912 (87.5%)	274 (12.5%)	
30~ 39	2757 (90.8%)	278 (9.2%)	
40~ 49	2094 (90.5%)	220 (9.5%)	
50~ 59	1459 (87.8%)	203 (12.2%)	
60~ 69	793 (89.4%)	94 (10.6%)	
≥ 70	494 (79.2%)	130 (20.8%)	
Blood pressure			0.02
Normotensive ^b^	7260 (89.2%)	879 (10.8%)	
SBP < 110 mmHg	6424 (88.8%)	814 (11.2%)	
SBP < 100 mmHg	2249 (87.5%)	320 (12.5%)	
SBP < 95 mmHg	942 (86.3%)	149 (13.7%)	
SBP < 90 mmHg	266 (83.4%)	53 (16.6%)	
BMI (mean), kg/m^2^	22.6 ± 3.1	22.7 ± 3.6	0.50
BMI, kg/m^2^			0.01
< 18.5	672 (85.9%)	110 (14.1%)	
≥ 18.5, < 25	6871 (89.3%)	825 (10.7%)	
≥ 25	1947 (88.1%)	262 (11.9%)	
Total cholesterol (mean), mg/ml	184.2 ± 34.4	184.7 ± 35.6	0.66
House income			0.00
Elementary school	2137 (84.7%)	387 (15.3%)	
Middle school	2307 (88.6%)	297 (11.4%)	
High school	2418 (90.6%)	252 (9.4%)	
College/University	2564 (91.1%)	249 (8.9%)	
Educational level			0.00
Low	1049 (80.0%)	262 (20.0%)	
Low to intermediate	721 (86.9%)	109 (13.1%)	
Intermediate to high	3767 (89.2%)	458 (10.8%)	
High	3970 (91.5%)	370 (8.5%)	
Marital status			0.00
Single	2110 (86.2%)	338 (13.8%)	
Married	6818 (90.0%)	756 (10.0%)	
Rejection response	571 (84.6%)	104 (15.4%)	
Current smoke			0.01
Yes	1836 (87.1%)	271 (12.9%)	
No	7671 (89.2%)	928 (10.8%)	
Alcohol intake			0.02
1 drink</month	3457 (89.0%)	427 (11.0%)	
1~ 4 drink /month	3565 (89.5%)	418 (10.5%)	
≥ 2~ 3 drink /week	1550 (87.1%)	230 (12.9%)	
Depressed mood			0.00
Yes	701 (54.3%)	590 (45.7%)	
No	8807 (93.5%)	609 (6.5%)	
Stress perception			0.00
Yes	2182 (75.8%)	695 (24.2%)	
No	7327 (93.6%)	504 (6.4%)	
Diabetes mellitus			0.01
Yes	271 (83.9%)	52 (16.1%)	
No	9238 (89.0%)	1147 (11.0%)	
Stroke			0.00
Yes	33 (71.7%)	13 (28.3%)	
No	9476 (88.9%)	1186 (11.1%)	
MI/Angina pectoris			0.04
Yes	93 (82.3%)	20 (17.7%)	
No	9416 (88.9%)	1179 (11.1%)	
Depression			0.00
Yes	84 (50.0%)	84 (50.0%)	
No	9425 (89.4%)	1115 (10.6%)	

*SBP* Systolic Blood Pressure, *BMI* Body Mass Index

^a^Reference: No suicidal ideation. ^b^ Normotensive: 100 ≤ SBP < 120 mmHg & DBP < 80 mmHg

### Outcome data, main results

The association between low BP and suicidal ideation is shown in Table 3. In the hypotensive groups with different SBP cut-offs used to define low BP, the odds ratios (ORs) of suicidal ideation were compared with those of the normotensive group. Factors assumed to be related to both low BP and suicidal ideation were included as potential confounding variables. Thus sex, age, BMI, total cholesterol level, sociodemographic variables, such as household income, educational level, marital status, and lifestyle (current smoking status and alcohol intake), and medical conditions (DM, stroke, MI/angina pectoris, depression) were adjusted for in the analyses. Medication histories for those diseases were also considered, but they were excluded due to multi-collinearity with the corresponding diseases. All variables were identified as true confounders except for total cholesterol level. Although total cholesterol level was not correlated with suicidal ideation, it was left in the covariate models because it was lower in the hypotensive group of our study population, and other studies have suggested a correlation with suicide [25]. The results from five multiple logistic regression models (models I–V) differed in the potential confounding variables included. Multi-collinearity among the covariates in the models was not observed. Sex and age showed a major difference between the normotensive and hypotensive groups; thus, raising doubt regarding the effect modifications by interactions between sex and age, sex and BP, or age and BP. However, only an interaction between age and sex was identified. The interaction between sex and age was included in models IV and V.

**Table 3:** Association of low blood pressure with suicidal ideation in four different cut-off levels for low blood pressure

		Low blood pressure criteria			
		SBP < 110 mmHg	SBP < 100 mmHg	SBP < 95 mmHg	SBP < 90 mmHg
		Odds ratio (95% CI)			
Crude Model		1.07 (0.91 to 1.26)	1.33 (1.14 to 1.56)^***^	1.57 (1.28 to 1.93)^***^	2.07 (1.43 to 2.98)^**^
	Covariates				
Model I	Age, Sex, BMI, Total cholesterol level	1.01 (0.85 to 1.20)	1.26 (1.06 to 1.49)^**^	1.44 (1.17 to 1.78)^***^	1.87 (1.24 to 2.65)^**^
Model II	Model I + Household income, Educational level, Marital status	1.10 (0.92 to 1.32)	1.34 (1.13 to 1.59)^***^	1.50 (1.21 to 1.87)^***^	1.81 (1.26 to 2.77)^**^
Model III	Model II + Current smoking status, Alcohol intake	1.07 (0.89 to 1.29)	1.35 (1.13 to 1.62)^***^	1.51 (1.19 to 1.90)^***^	1.81 (1.17 to 2.77)^**^
Model IV	Model III + Sex*Age	1.03 (0.85 to 1.24)	1.29 (1.08 to 1.55)^**^	1.44 (1.14 to 1.82) ^**^	1.71 (1.11 to 2.62) ^*^
Model V	Model IV + DM, Stroke, MI/Angina pectoris, Depression	1.04 (0.86 to 1.26)	1.25 (1.04 to 1.50)^*^	1.43 (1.13 to 1.81) ^**^	1.74 (1.14 to 2.68) ^*^

Asterisks indicate statistical significance (*p < 0.05, **p < 0.01, *** *p* < 0.001)

Reference is normal blood pressure (100 ≤ SBP < 120 mmHg & DBP < 80 mmHg) group

*SBP* Systolic Blood Pressure, *BMI* Body Mass Index, *DM* Diabetes Mellitus, *MI* Myocardial infarction

Compared with the normotensive reference group, the ORs for suicidal ideation were significantly higher in the SBP < 100, < 95, and < 90 mmHg hypotensive groups (OR = 1.29, 95% CI, 1.08 to 1.55; OR = 1.44, 95% CI, 1.14 to 1.82; and OR = 1.71, 95% CI, 1.11 to 2.62 for SBP < 100, SBP < 95, and SBP < 90 mmHg, respectively) after adjusting for sex, age, BMI, total cholesterol level, household income, educational level, marital status, current smoking status, alcohol intake, and the interaction between sex and age (model IV). Adding the clinical morbidities of DM, stroke, MI/angina, and depression as covariates had little effect on the strength of the associations (OR = 1.25, 95% CI, 1.04 to 1.50; OR = 1.43, 95% CI, 1.13 to 1.81; and OR = 1.74, 95% CI, 1.14 to 2.68 for SBP < 100, < 95, and < 90 mmHg, respectively; model V). No association was observed in the SBP < 110 mmHg hypotensive group.

### Supplementary analyses

Both the prehypertensive and hypertensive groups were also examined to see whether those BPs were associated with suicidal ideation according to the multivariate logistic regression. In contrast to the low BP group, no significant associations were shown (OR = 1.03, 95% CI, 0.87 to 1.23, OR = 1.05, 95% CI, 0.88 to 1.27 in the prehypertensive and hypertensive groups, respectively; model V). An additional table file shows this in more detail [see Additional file 1].

ORs and CIs for suicidal ideation in four different cut-off levels for low BP, prehypertension, and hypertension compared to normal BP are shown together in the figure (models IV and V) [see Additional file 2].

Overall, the lower the BP, the higher the risk for suicidal ideation among the hypotensive groups. However, no significant differences were observed in the risk of suicidal ideation among the higher BP (SBP < 110 mmHg hypotensive criteria, pre-hypertensive and hypertensive) groups and the normotensive group.

## Discussion

This population-based study showed an association between low BP and suicidal ideation in a large general population representing Korean adults. This is consistent with several earlier studies supporting a correlation between low BP and psychological symptoms, such as depression and anxiety, even though outcome variables are distinct [9, 26, 27]. In this study, four cut-off values were used to define low BP: SBP < 110, < 100, < 95, and < 90 mmHg. All of these hypotensive groups except the SBP < 110 mmHg group showed meaningful correlations with suicidal ideation. As this is the first report to explore the relationship between low BP and suicidal ideation, it was not possible to compare our results with those of other studies assessing the same outcome. Instead, we compared our results with those of Hildrum et al. [9], who evaluated the correlation between low BP and depression and anxiety, using a similar study design. ORs for the comorbidities of anxiety and depression in the lowest fifth percentile of SBP in their study were similar with ORs for suicidal ideation in the SBP < 95 mmHg group in our study; the proportion of subjects corresponding to the lowest fifth percentile of SBP in Hildrum et al. is similar to those in the SBP < 95 mmHg group in this study, suggesting that low BP might affect suicidal ideation to similar extent as it affects typical psychiatric symptoms.

Because there is little research on the factors that influence low BP, we used five multiple regression models including different covariates to assess the effects of the potential confounding variables more closely. The analyses showed stronger correlations in models II-V, which included more potential confounding variables compared with Model I, in which only basic adjustments were made for sex, age, BMI, and total cholesterol level. Not presented in the results here, but in an additional multivariate model that included occupation, LDL-cholesterol level, and hemoglobin level, this association was still significant. These additional findings further support a correlation between low BP and suicidal ideation. We adjusted for several major diseases, such as DM, stroke, MI/angina pectoris, and depression, with little change in the outcome (models IV and V). Other underlying diseases may be confounding variables, but a previous study suggested no difference in baseline diseases between subjects with hypotension and those with a normal BP. A longitudinal follow-up study of 1337 patients conducted between 1958 and 1999 revealed no differences in underlying diseases between patients with low BP and those with normal BP, including malignant diseases, pulmonary diseases, gastric ulcers, mental disorders, blindness, and valvular heart diseases, except anemia [28]. Medication of antidepressants, antipsychotics, anticonvulsants, antihypertensives, and others may be potential compounding variables. Among these, antidepressants, antidiabetes, treatment for stroke and MI/angina pectoris were available in KNHANES data and considered. However, multi-collinearity with the corresponding diseases was apparent and excluded from the final analysis. Subjects who were using antihypertensive medications were included among those defined as having hypertension.

There is no consensually accepted definition for low BP. Most experts consider SBP ≤ 90 mmHg and/or DBP ≤ 60 mmHg as hypotensive [29]. The World Health Organization defines low BP as SBP < 110 in men and SBP < 100 mmHg in women, regardless of DBP [30], whereas a German study stated that values of 100/60 mmHg are hypotensive [31] (quoted in [32]). Some studies have used various criteria, such as SBP < 120 or DBP < 75 (for elderly subjects) [4], SBP < 110 [2], and SBP < 100 mmHg [3], whereas other studies have used the lowest 5th centile [9] or tertile [6]. These different standards for low BP make it even more difficult for comparisons and evaluations of the effects of low BP. The present study offers a methodological advantage in this regard, as the relationship between low BP and suicidal ideation was checked for each SBP category (< 110, < 100, < 95, and < 90 mmHg), which allowed us to identify changes in the results according to different BP cut-off values and to verify which BP level was clinically significant. Patients with the lower BP cut-off showed a more pronounced tendency toward suicidal ideation in all models, suggesting the possibility that lower BP is associated with suicidal ideation. The OR for SBP < 110 mmHg did not differ from that for normal BP for suicidal ideation. In contrast, the lower BP groups, with cut-off values of SBP < 100, < 95, and < 90 mmHg, showed significantly higher levels of suicidal ideation in all covariate models, suggesting that SBP < 100 mmHg is relevant to the relationship between low BP and suicidal ideation. A considerable number of people (11% of the adult population in Korea) have SBP < 100 mmHg. The association between suicidal ideation and this BP level raises the assumption that not only quite low but relatively low BP can be associated with suicidal ideation.

A large size sample that represents the general population of adults is another strength of this study. Although the prevalence of low BP is higher in younger people [3, 33], the majority of studies on the association between low BP and psychiatric disturbances have paid attention to specific age groups [34, 35], particularly to the elderly [4, 7, 26, 27, 36], making it impossible to know the influence throughout all ages of adults.

The results of this study can be generalized to all Korean adults, as the statistics reflect the complex sampling design, non-response to the survey, and post-stratification in the analyses. When weighted, the risk of suicidal ideation in the low BP group increased compared with the unweighted results (OR = 1.20, 95% CI, 1.02 to 1.40; OR = 1.22, 95% CI, 0.98 to 1.50; and OR = 1.39, 95% CI, 0.96 to 1.96 for SBP < 100, < 95, and < 90 mmHg, respectively; unweighted results, model V). It is assumed that the relationship between low BP and suicidal ideation would be more evident by applying weights during the analyses, as the sample reflected a higher population of younger people in urban areas than in rural areas.

There are no published studies investigating the impact of low BP on somatic or psychiatric symptoms in a general adult population in Asia, although the prevalence of hypotension is much higher in younger adults. This study is the first in Asia to investigate the relationship between low BP and substantive psychiatric symptoms in a large general population which includes young adults.

Unlike low BP, high BP was not associated with suicidal ideation in this study. Despite not adjusting for all comorbid cardiovascular diseases but several diseases, which are far more common in hypertensive patients and which affect mental health negatively [37], no association with suicidal ideation was seen in the hypertensive group, further reinforcing the suspicion that low BP itself is related to suicidal ideation. These results are consistent with previous studies showing no significant correlation between high BP and suicidal ideation [22, 23]. However, because a large-scale study suggested a high risk of suicidal ideation in patients with hypertension [21], further studies are needed.

In all five covariate models, the ORs in the prehypertensive group consistently did not differ from those in the normal BP group. These results further support that only low BP, among the different BP levels, is related to suicidal ideation. In addition, although prehypertension may be a risk factor for cardiovascular diseases and should be managed, it does not have a negative impact on mental health [see Additional file 1]. It is assumed that low BP poses quite different health issues from those of higher BP.

The results of this study are in accordance with the BP-emotional dampening hypothesis which suggests that BP has inhibitory effect on overall negative emotional experience and pain perception [38]. Baroreflex sensitivity (BRS) is strongly doubted as a potential mechanism [39]. In high BP, where baroreceptor stimulation dominates, increased BRS results in stronger cortical inhibition [39]. By contrast, in individuals with low BP, who predominantly exhibit baroreceptor inhibition, high BRS is accompanied by reduced central nervous inhibition and thus increased cortical arousal [39]. In several studies supporting this hypothesis, higher BP was associated with dampened responses to negative emotional stimuli [40]. On the contrary, high level of anxiety, hostility, and worry were clearly prevalent in people with low BP who were prevailed with reduced BRS [38, 41]. Psychophysiological approaches to explore whether BRS mediates the link between low BP and suicidal ideation will help to understand the mechanism.

The biological mechanism for the relationship between low BP and negative health effects is not well established. However, studies have suggested that reduced cerebral perfusion in a patient with low BP might be related with depression. Reduced microvascular circulation and oxygen transfer have been hypothesized to be a cause for the physical fatigue in a patient with a low BP. Erythrocyte velocity decreased at very low BP (< 70 mmHg) in a capillary dynamics study using TV microscopy at different BP levels [42]. The hypothesis that inappropriate energy production and accumulation of metabolites are causes of physical fatigue has also been suggested; [32] However, no evidence supports it. Conversely, studies suggesting that depression causes low BP have indicated that overexpressed neuropeptide Y in a patient with low BP is likely to mediate depression [13, 43]. More in-depth biological studies are needed to explain the mechanism.

Although it is clear that mental disorders, such as depression, are related to suicidal ideation, physical conditions are not. The present study is the first to investigate the association between low BP and suicidal ideation, which is a concrete indicator of a negative psychiatric state, thus driving the need to re-evaluate the health implications of low BP. Mental health conditions including suicidal ideation needs to be carefully monitored among those with low BP.

### Limitations

Our study had some limitations. First, it was cross-sectional, so a two-way relationship is possible. Although Paterniti et al. [27] showed in a longitudinal study that baseline high depressive symptomatology was not a risk factor for low BP, supporting the low likelihood of a reverse correlation, subsequent prospective studies have found a reverse association between low BP and depression [13, 44, 45]. It is possible that depression may result in low BP by way of weight loss and reduced activities [8]. Although the likelihood of suicidal ideation to induce low BP seems intuitively low, future prospective studies should be conducted to investigate causality. Second, although the quality of the information collected from the hypotensive group, which is normally regarded as normal BP, was not expected to be much different from that of the normotensive group, the possibilities of information bias and recall bias cannot be ruled out, as this study used survey data. Third, the questions pertaining to suicidal ideation used in this study might not to be a sufficient assessment. However, the questionnaire used to screen for suicidal ideation followed the definition of suicidal ideation [46] and considering that the proportion with suicidal ideation in this study was similar to those in other studies [47], the results are considered reliable. Finally, in this study, we only used the levels of SBP to define low BP, and did not consider DBP. Because some previous studies showed different outcomes depending on whether the cut-off values to define hypotension are based on SBP or DBP [9, 48], it is also necessary to explore how suicidal ideation is related to low DBP.

## Conclusions

Although many studies suggest that low BP is associated with neuropsychological problems, including depression, anxiety, cognitive dysfunction, and dementia, no studies have investigated the association between low BP and suicidal ideation. We found that low SBP is associated with suicidal ideation in a general population. The association was significant for low BP defined by a SBP < 100 mmHg, and the strength of the association increased as the strictness of the criteria for low BP increased. This significant association seen in hypotension was not present in the hypertensive or prehypertensive groups but was hypotension-specific. This study has the advantage of being a large general population-based study covering a wide range of ages and using different cut-off levels for low BP, so we could examine whether there was a quantitative relationship in which the risk of suicidal ideation increases as the cut-off level of low BP is lower.

## Additional files

Additional file 1: Table S1. Association of high blood pressure with suicidal ideation, crude and five multiple covariate models. Both prehypertensive and hypertensive group were also examined to see whether those blood pressure have associations with suicidal ideation by using multivariate logistic regression. In contrast to low BP group, no significant associations were shown in all covariates models. (DOCX 18 kb)

Additional file 2: Figure S1. Association of blood pressure with suicidal ideation in four different cut-off levels for low BP, prehypertension and hypertension in multiple logistic regression model IV and model V. ORs and confidence intervals for suicidal ideation in four different cut-off levels for low BP, prehypertension and hypertension comparing to normal blood pressure were shown together in the figure (model IV and V) [see Additional file 2]. In overall, the lower the blood pressure, the higher the risk of suicidal ideation among the hypotensive groups. However, there was no statistically significant differences in the risk of suicidal ideation among the higher blood pressure (pre-hypertensive and hypertensive) groups and normotensive group. (PDF 165 kb)

## Abbreviations

BMI: Body Mass Index
BP: Blood pressure
CI: Confidence interval
DBP: Diastolic blood pressure
DM: Diabetes Mellitus
KNHANES: Korean National Health and Nutrition Examination Survey
MI: Myocardial Infarction
OR: Odds ratio
SBP: Systolic blood pressure
