# Supplementary material for: Association of low blood pressure with suicidal ideation: a cross-sectional study of 10,708 adults with normal or low blood pressure in Korea
Source: BMC Public Health. 2018 Mar 1;18:200. doi: 10.1186/s12889-018-5106-5 (PMC5831223; doi:10.1186/s12889-018-5106-5)
Supplement: Supplementary file 1 — Association of high blood pressure with suicidal ideation, crude and five multiple covariate models. Both prehypertensive and hypertensive group were also examined to see whether those blood pressure have associations with suicidal ideation by using multivariate logistic regression. In contrast to low BP group, no significant associations were shown in all covariates models. (DOCX 18 kb) [file 12889_2018_5106_MOESM1_ESM.docx]

Supplement Table 1. Association of high blood pressure with suicidal ideation, crude and 5 multiple covariate models.

|  |  | Prehypertension ^a^ | Hypertension ^b^ |
| --- | --- | --- | --- |
|  |  | Odds ratio (95% CI) Odds ratio (95% CI) | |
|  | Crude | 1.05 (0.92 to 1.20) | 1.49 (1.32 to 1.69)*** |
|  | Covariates |  |  |
| Model Ⅰ | Age, Sex, BMI, Total cholesterol level | 1.01 (0.86 to 1.18) | 1.18 (1.01 to 1.38) ** |
| Model Ⅱ | Model Ⅰ + Household income, Educational level, Marital status | 1.00 (0.85 to 1.17) | 1.08 (0.92 to 1.28) |
| Model Ⅲ | Model Ⅱ + Current smoking status, Alcohol intake | 1.03 (0.87 to 1.22) | 1.08 (0.90 to 1.29) |
| Model Ⅳ | Model Ⅲ + Sex*Age | 1.05 (0.89 to 1.25) | 1.09 (0.91 to 1.31) |
| Model Ⅴ | Model Ⅳ + DM, Stroke, MI/angina pectoris, Depression | 1.03 (0.87 to 1.23) | 1.05 (0.88 to 1.27) |

Asterisks indicate statistical significance (***p*<0.01, *** *p*<0.001)

Reference is normal blood pressure (100 ≤ SBP < 120 mmHg & DBP < 80 mmHg) group

^a^ Prehypertension: 120 ≤ SBP < 140 mmHg or 80 ≤ DBP < 90 mmHg

^b^ Hypertension: SBP ≥ 140 mmHg or DBP ≥ 90 mmHg

DM, Diabetes Mellitus; MI, Myocardial infarction.
