# Supplementary figures and images for: Association of low blood pressure with suicidal ideation: a cross-sectional study of 10,708 adults with normal or low blood pressure in Korea
Source: BMC Public Health. 2018 Mar 1;18:200. doi: 10.1186/s12889-018-5106-5 (PMC5831223; doi:10.1186/s12889-018-5106-5)

**Model IV**

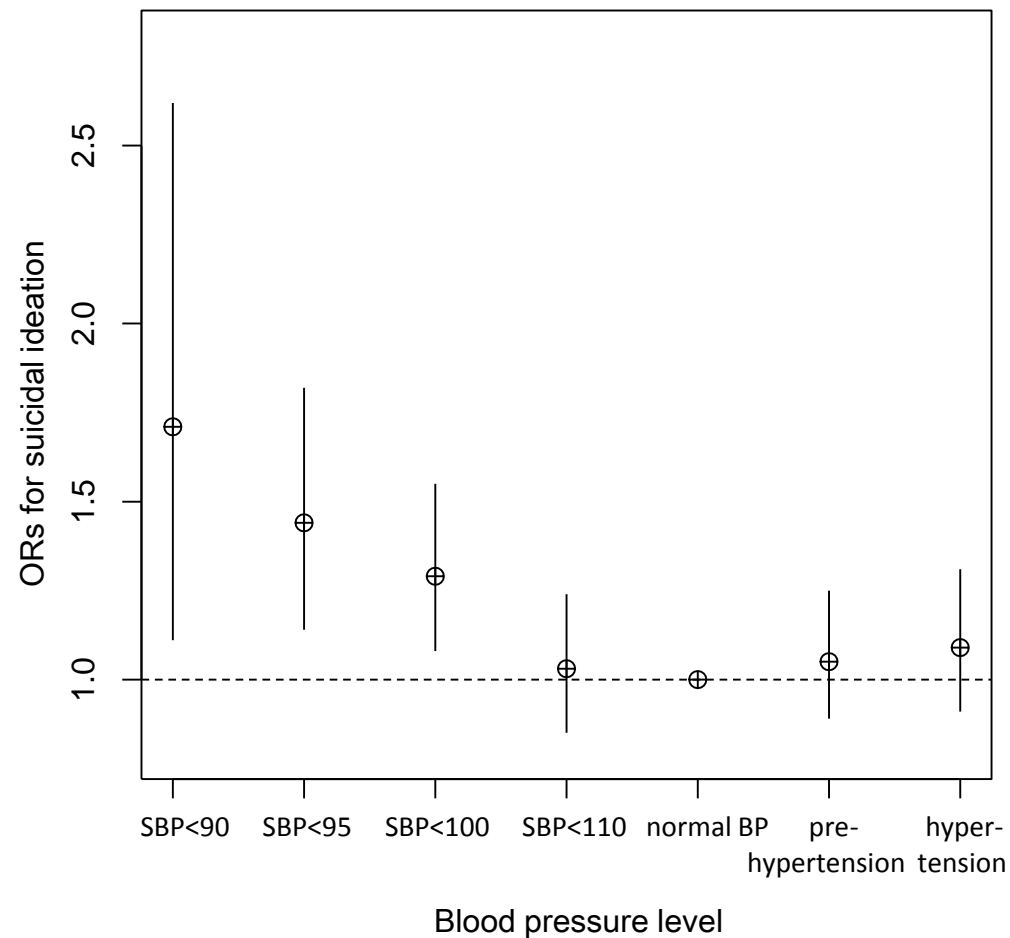

**Model V**

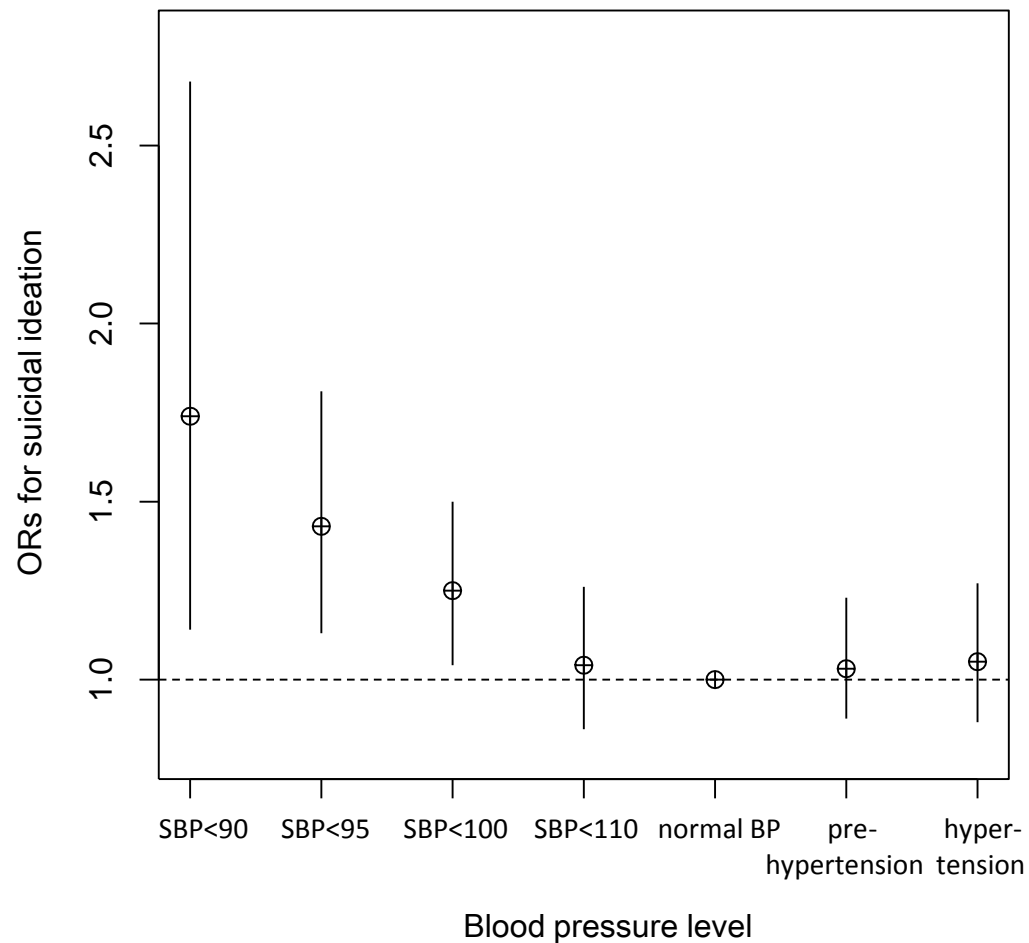

Supplement: Supplementary file 2 — Association of blood pressure with suicidal ideation in four different cut-off levels for low BP, prehypertension and hypertension in multiple logistic regression model IV and model V. ORs and confidence intervals for suicidal ideation in four different cut-off levels for low BP, prehypertension and hypertension comparing to normal blood pressure were shown together in the figure (model IV and V) [see Additional file 2]. In overall, the lower the blood pressure, the higher the risk of suicidal ideation among the hypotensive groups. However, there was no statistically significant differences in the risk of suicidal ideation among the higher blood pressure (pre-hypertensive and hypertensive) groups and normotensive group. (PDF 165 kb) [file 12889_2018_5106_MOESM2_ESM.pdf]
